# Supplementary material for: Effectiveness of a Video-Based Intervention on Reducing Perceptions of Fear, Loneliness, and Public Stigma Related to COVID-19: A Randomized Controlled Trial
Source: Int J Public Health. 2021 Aug 17;66:1604164. doi: 10.3389/ijph.2021.1604164 (PMC8407346; doi:10.3389/ijph.2021.1604164)
Supplement: Supplementary file 2 [file DataSheet1.pdf]

Supplementary Material: Appendices, Tables and Figures for

"Effectiveness of a Video-Based Intervention on Reducing Perceptions of Fear, Loneliness, and Public Stigma Related to COVID-19: A Randomized Controlled Trial"

Authors: Linda Valeri, Doron Amsalem, Samantha, Jankowski, Ezra Susser, and Lisa Dixon.

Appendix 1

Baseline Questionnaire, Version 2, 3/26/20

1. How old are you? _____

2. Are you Hispanic or Latino?

   a. Yes
   b. No
   c. Prefer not to answer

3. What is your race or ethnicity?

   a. African American
   b. Asian
   c. Native American
   d. White
   e. Other, please specify ____
   f. Prefer not to answer

4. What is your gender identity?

   a. Male
   b. Female
   c. Transgender
   d. Other, please specify____
   e. Prefer not to answer

Pre-, post-, and follow-up questionnaire

Currently, the world is experiencing a Coronavirus (COVID-19) outbreak, which is spreading in the United States and has been recently recognized as a pandemic. The following questions will examine thoughts and perceptions regarding COVID-19.

1. How much time per day do you spend on average connected to the internet or other media?

a. Less than one hour
b. 1-4 hours
c. 4-7 hours
d. More than 7 hours

2. How many times did you contact (in person or via video call) your friends/family members over the last week?

a. 0-2
b. 2-5
c. 5-7
d. More than 7
e. Prefer not to answer

3. Do you know anyone who was or is currently quarantined?

a. No
b. Yes – me
c. Yes – friend/family member
d. Prefer not to answer

If they say "Yes – me" the following question will appear:

3A. Have you been diagnosed with Coronavirus (COVID-19)?

a. No
b. Yes
c. Prefer not to answer

If they say "Yes – friend/family member", the following question will appear:

3B. Have they been diagnosed with Coronavirus (COVID-19)?

a. No
b. Yes
c. Prefer not to answer

4. How much are you afraid of the consequences of the Coronavirus (COVID-19) outbreak?

a. Not at all
b. A bit
c. Quite a bit
d. A lot

5. How much are you afraid to be diagnosed with Coronavirus (COVID-19)?

a. Not at all
b. A bit
c. Quite a bit
d. A lot

6. How much are you afraid to be isolated because of Coronavirus (COVID-19)?

a. Not at all
b. A bit
c. Quite a bit
d. A lot

7. How lonely do you feel?

a. Not at all
b. A bit
c. Quite a bit
d. A lot

8. How much are you afraid of people diagnosed with Coronavirus (COVID-19)?

a. Not at all
b. A bit
c. Quite a bit
d. A lot

9. I worry that elderly people may not be getting adequate treatment for Coronavirus (COVID-19)

a. Strongly disagree
b. Disagree
c. Agree
d. Strongly agree
e. Prefer not to answer

10. It’s easier to avoid friends than worry about telling someone about having Coronavirus (COVID-19)

a. Strongly disagree
b. Disagree
c. Agree
d. Strongly agree
e. Prefer not to answer

11. People feel guilty because they have Coronavirus (COVID-19)

a. Strongly disagree
b. Disagree
c. Agree
d. Strongly agree
e. Prefer not to answer

12. I worry that people may judge me when they learn I have Coronavirus (COVID-19)

a. Strongly disagree
b. Disagree
c. Agree
d. Strongly agree
e. Prefer not to answer

13. People with Coronavirus (COVID-19) never feel the need to hide the fact that they have Coronavirus (COVID-19)

a. Strongly disagree
b. Disagree
c. Agree
d. Strongly agree
e. Prefer not to answer

Appendix 2

Vignette- Version 1, 3/13/20

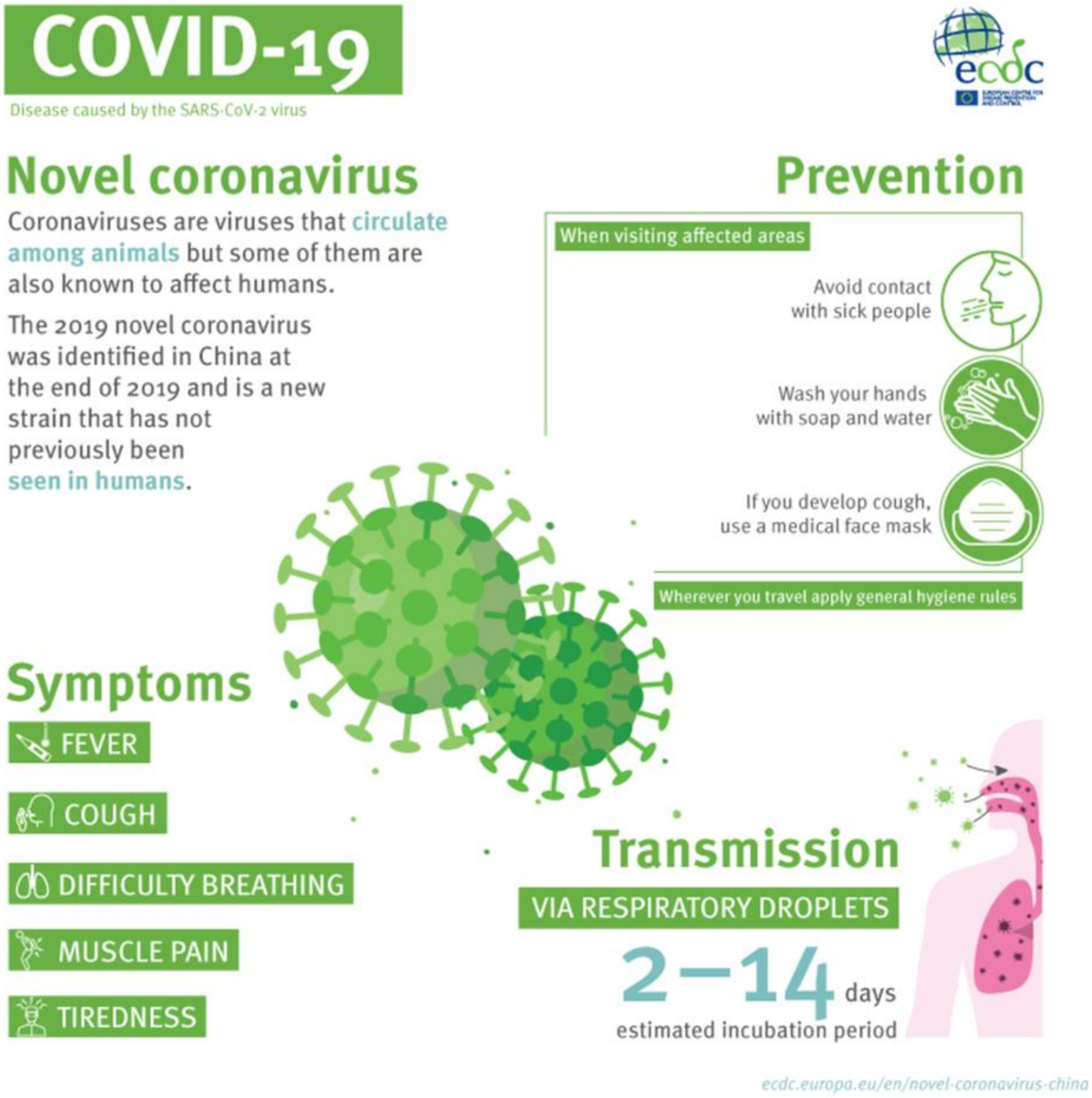

Appendix 3

Video Interventions

https://youtu.be/fuua3nDgCkU

and

https://youtu.be/kxNqToyITcQ

Video-solo Transcript:

Sophia:

Hi guys, some people asked me to upload a video so I can share how it is to be in isolation since I have got the coronavirus. So [coughs], I'm mostly okay. Yeah I know, I have enough food and I have enough Netflix, of course. [Coughs]. My symptoms are pretty mild, so it feels like I've got a flu.

Um, to be honest the hardest part is that it feels like people are avoiding me, and I'm not sure if it's because they think they can get the virus from my phone calls or they're just afraid I'm gonna ask them for some favors or to stop by or anything.

Um, I'm afraid that some of them might be angry with me because I exposed them to the virus. And in that case, I'm sorry. I just couldn't possibly know that I'm doing that. I just… um, I'm tired of screens and I think that I need some human interaction. It could have happened to anyone. So…

Video-Friend Transcript:

Sophia: Hey!

Friend: Hey, Sophia.

Sophia: How are you?

Friend: Good! How are you?

Sophia: [Laughs]. I'm okay, I'm okay. I think…

Friend: Yeah.

Sophia: How is it for you to be at home without going out? I heard that you can't go out now.

Friend: Yeah, I think we all cannot go out. It's not easy, but you know I'm holding on. Um, living it day to day. I think it's ok. Yeah.

Sophia: And how are your parents?

Friend: My parents are fine. I’m calling every day. I am a little bit concerned regarding my father, you know, he’s sick. You know. Uh, I’m trying to convince him not to leave home at all, but it’s not easy. He needs his like breaks and to go to the supermarket and buy stuff. He’s insisting on it. Both my mom and me are trying to convince him not to do it, but it’s not easy. You know him.

Sophia: Yeah, yeah, I know.

Friend: Well, you should just talk more with your family. How is your family doing?

Sophia: Well, my family is ok. Um, my parents are, are, you know, they think that they’re big heroes and that nothing will happen to them, because you know they went through wars so they think it’s something they shouldn’t be worried about. So they do everything as usual. They go to their jobs and to the supermarket, they’re not afraid. I don’t know. I can’t convince them otherwise. I’m trying to. Is it so easy and natural for you to communicate with people through the screen?

Friend: You know, it’s not the same. But I would say, first of all, it’s much better than I assumed. Uh, and in a weird way I also contact more, you know my relatives and friends because you know, I’m worried. So I do speak more with them than I used to speak before it started and you know obviously it’s not the same, but it’s you know, good enough, I would say.

Sophia: Yes.

Table S.1 Baseline characteristics by missing follow-up status

	Missing Follow-up (N=360)	Not Missing Follow-up (N=628)	Total (N=988)
**Age (years)**			
Mean (SD)	34.8 (11.4)	38.4 (11.9)	37.1 (11.9)
Median [Min, Max]	32.0 [18.0, 70.0]	36.0 [18.0, 70.0]	34.0 [18.0, 70.0]
**Risk Level**			
High Risk	104 (28.9%)	189 (30.1%)	293 (29.7%)
Low Risk	256 (71.1%)	439 (69.9%)	695 (70.3%)
**Gender**			
Male	215 (59.7%)	375 (59.7%)	590 (59.7%)
Female	144 (40.0%)	246 (39.2%)	390 (39.5%)
Transgender	1 (0.3%)	1 (0.2%)	2 (0.2%)
Other	0 (0%)	2 (0.3%)	2 (0.2%)
Missing	0 (0%)	4 (0.6%)	4 (0.4%)
**Race/Ethnicity**			
African American	36 (10.0%)	52 (8.3%)	88 (8.9%)
Asian	29 (8.1%)	59 (9.4%)	88 (8.9%)
Native American	11 (3.1%)	6 (1.0%)	17 (1.7%)
White	275 (76.4%)	487 (77.5%)	762 (77.1%)
Prefer not to answer	2 (0.6%)	7 (1.1%)	9 (0.9%)
Other	7 (1.9%)	17 (2.7%)	24 (2.4%)
**Social contact past week**			
0-2	75 (20.8%)	155 (24.7%)	230 (23.3%)
2-5	144 (40.0%)	206 (32.8%)	350 (35.4%)
5-7	47 (13.1%)	111 (17.7%)	158 (16.0%)
More than 7	92 (25.6%)	155 (24.7%)	247 (25.0%)
Missing	2 (0.6%)	1 (0.2%)	3 (0.3%)
**Time on internet past week**			
Less than one hour	5 (1.4%)	6 (1.0%)	11 (1.1%)
1-4 hours	82 (22.8%)	119 (18.9%)	201 (20.3%)
4-7 hours	136 (37.8%)	237 (37.7%)	373 (37.8%)
More than 7 hours	137 (38.1%)	266 (42.4%)	403 (40.8%)
**Quarantine status**			
No	198 (55.0%)	398 (63.4%)	596 (60.3%)
Yes - me	68 (18.9%)	106 (16.9%)	174 (17.6%)
Yes - friend/family member	89 (24.7%)	123 (19.6%)	212 (21.5%)
Missing	5 (1.4%)	1 (0.2%)	6 (0.6%)

Table S.2 Longitudinal intervention effects on continuous stigma and fear score adjusting for drop-out using inverse probability of censoring weights adjusting for baseline covariates (age, quarantine status, gender, risk, race, baseline score).

	Stigma score			Fear score		
Predictors	*Estimates*	*CI*	*p*	*Estimates*	*CI*	*p*
time	-1.01	-1.12 – -0.89	**<0.001**	-0.07	-0.16 – 0.02	0.139
Video.Solo vs Informational sheet	-1.07	-1.44 – -0.70	**<0.001**	0.20	-0.09 – 0.50	0.174
Video. Friend vs Informational sheet	-0.94	-1.36 – -0.51	**<0.001**	-0.04	-0.37 – 0.30	0.821
time * Video.Solo vs Informational sheet	0.33	0.18 – 0.48	**<0.001**	0.02	-0.10 – 0.14	0.749
time * Video. Friend vs Informational sheet	0.19	0.01 – 0.37	0.041	0.00	-0.15 – 0.15	0.989

Table S.3 Longitudinal intervention effects on ordinal items loneliness and fear of COVID+ people adjusting for drop-out using inverse probability of censoring weights adjusting for baseline covariates (age, quarantine status, gender, risk, race, baseline score).

	Fear of people COVID+			Loneliness		
Predictors	*Estimates*	*CI*	*p*	*Estimates*	*CI*	*p*
time	0.89	0.81 – 0.97	**<0.001**	0.85	0.75 – 0.95	0.020
Video.Solo vs Informational sheet	0.87	0.51 – 1.48	0.629	0.50	0.25 – 1.00	0.051
Video. Friend vs Informational sheet	0.91	0.54 – 1.52	0.721	0.23	0.10 – 0.46	**<0.001**
time * Video.Solo vs Informational sheet	0.70	0.60 – 0.82	**<0.001**	1.01	0.83 – 1.22	0.877
time * Video. Friend vs Informational sheet	0.74	0.63 – 0.87	**<0.001**	1.10	0.92 – 1.32	0.276

Table S.4 Longitudinal intervention effects comparing video groups to informational sheet group on secondary outcomes adjusting for covariates (age, quarantine status, gender, risk, race, baseline score.)

	**Internet use**			**Social contacts**		
Predictors	*Estimates*	*CI*	*p*	*Estimates*	*CI*	*p*
time	0.87	0.75 – 1.01	0.081	1.01	0.90 – 1.14	0.663
Video.Solo vs Informational sheet	1.41	0.51 – 1.48	0.442	1.99	1.09 – 3.64	0.024
Video. Friend vs Informational sheet	1.73	0.54 – 1.52	0.259	0.88	0.48 – 1.61	0.695
time * Video.Solo vs Informational sheet	1.03	0.60 – 0.82	0.759	0.93	0.78 – 1.12	0.477
time * Video. Friend vs Informational sheet	0.93	0.63 – 0.87	0.554	1.03	0.85 – 1.24	0.730

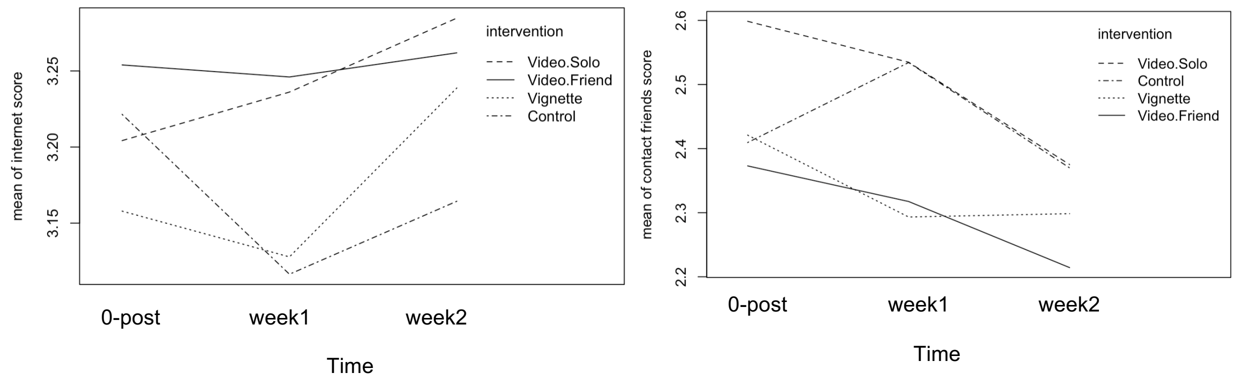

Figure S.1 Trajectory of time spent on internet and social contact scores by intervention group post intervention on April 2 (time =0-post), follow-up 1on April 9 (week1), follow-up 2 on April 16 (week2).
